# Supplementary material for: The Atypical Chemerin Receptor GPR1 Displays Different Modes of Interaction with β-Arrestins in Humans and Mice with Important Consequences on Subcellular Localization and Trafficking
Source: Cells. 2022 Mar 18;11(6):1037. doi: 10.3390/cells11061037 (PMC8947326; doi:10.3390/cells11061037)
Supplement: Supplementary file 1 [file cells-11-01037-s001.zip › cells-1605111-supplementary.pdf]

Supplementary Materials

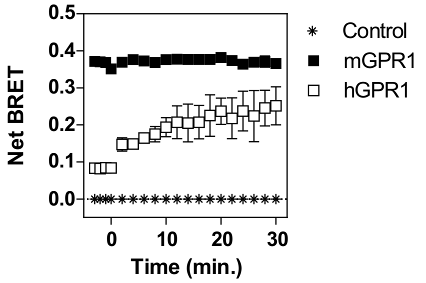

Figure S1 Real-time measurement of BRET signal in HEK293T cells expressing rat β-arrestin2-*R*Luc in combination with hGPR1-Venus or mGPR1-Venus , in basal conditions and after stimulation with 100 nM chemerin. Results are expressed as Net BRET corresponding to the difference between the BRET signal measured between the donor and the acceptor pair and the BRET signal measured with the donor only. Data represent the mean ± S.E.M. of two independent experiments.

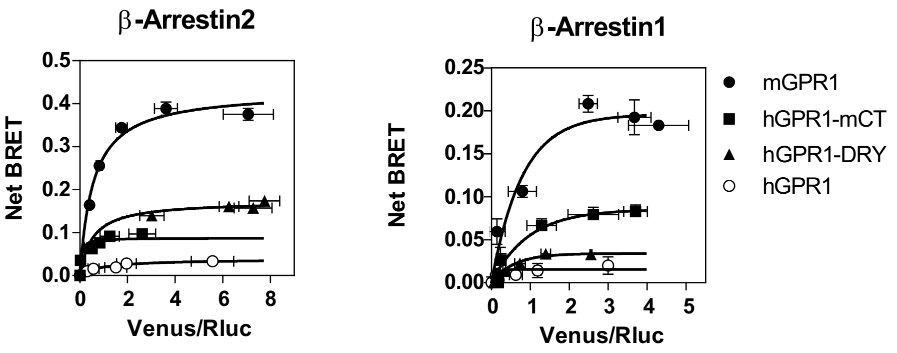

Figure S2. $R^{3.50}$ and the C-terminus of mGPR1 are involved in its interaction with β-arrestins. A-B BRET titration curves obtained with HEK293T cells transfected with a constant amount of β-arrestin2-*R*Luc (A) or β-arrestin1-*R*Luc (B) and increasing amounts of hGPR1-Venus, hGPR1-DRY-Venus, hGPR1-mCT or mGPR1-VenusResults are expressed as Net BRET corresponding to the difference between the BRET signal measured between the donor and the acceptor pair and the BRET signal measured with the donor only. Data represent the mean ± S.E.M. of at least three independent experiments.

.
